# Molecular basis of targeted therapy in T/NK-cell lymphoma/leukemia: A comprehensive genomic and immunohistochemical analysis of a panel of 33 cell lines

**DOI:** 10.1371/journal.pone.0177524

**Published:** 2017-05-15

**Authors:** Rufino Mondejar, Cristina Pérez, Arantza Onaindia, Nerea Martinez, Julia González-Rincón, Helena Pisonero, Jose Pedro Vaqué, Laura Cereceda, Miguel Santibañez, Margarita Sánchez-Beato, Miguel Angel Piris

**Affiliations:** 1 Cancer Genomics Laboratory, Instituto de Investigación Marqués de Valdecilla, IDIVAL, Santander, Spain; 2 Pathology Department, Hospital Universitario Marqués de Valdecilla, Santander, Spain; 3 Lymphoma Research Group (Medical Oncology Service) Oncohematology Area, Instituto Investigación Sanitaria Puerta de Hierro-Majadahonda (IDIPHIM), Madrid, Spain; 4 Instituto de Biomedicina y Biotecnología de Cantabria, IBBTEC (CSIC, Universidad de Cantabria), Departamento de Biología Molecular, Universidad de Cantabria, Santander, Spain; 5 Universidad de Cantabria-IDIVAL, Santander, Spain; Seconda Universita degli Studi di Napoli, ITALY

## Abstract

T and NK-cell lymphoma is a collection of aggressive disorders with unfavorable outcome, in which targeted treatments are still at a preliminary phase. To gain deeper insights into the deregulated mechanisms promoting this disease, we searched a panel of 31 representative T-cell and 2 NK-cell lymphoma/leukemia cell lines for predictive markers of response to targeted therapy. To this end, targeted sequencing was performed alongside the expression of specific biomarkers corresponding to potentially activated survival pathways. The study identified *TP53*, *NOTCH1* and *DNMT3A* as the most frequently mutated genes. We also found common alterations in JAK/STAT and epigenetic pathways. Immunohistochemical analysis showed nuclear accumulation of MYC (in 85% of the cases), NFKB (62%), p-STAT (44%) and p-MAPK (30%). This panel of cell lines captures the complexity of T/NK-cell lymphoproliferative processes samples, with the partial exception of AITL cases. Integrated mutational and immunohistochemical analysis shows that mutational changes cannot fully explain the activation of key survival pathways and the resulting phenotypes. The combined integration of mutational/expression changes forms a useful tool with which new compounds may be assayed.

## Introduction

T and NK-cell leukemia/lymphoma is a collection of aggressive disorders with unfavorable outcome accounting for 10–15% of non-Hodgkin lymphomas. The most recent WHO Classification established 23 subtypes grouped by clinical presentation [1]. T-cell lymphomas (TCLs) are the most common group, and within this subgroup the major subtypes are peripheral TCL (PTCL), not otherwise specified (PTCL-NOS), angioimmunoblastic T cell lymphoma (AITL), anaplastic lymphoma kinase (ALK)-positive anaplastic large cell lymphoma (ALCL) and ALK-negative ALCL. Among these, PTCL-NOS is the most widespread subtype worldwide and typically represents a variant that does not meet the criteria for other subtypes [2]. On the other hand, T-cell acute lymphoblastic leukemia (T-ALL), a T-cell neoplasm of lymphoblasts, accounts for about 15% and 25% of acute lymphoblastic leukemia (ALL) cases in pediatric and adult cohorts, respectively.

Nowadays, PTCL diagnosis requires the integration of information about clinical status, morphology, immunohistochemistry, flow cytometry, cytogenetics and molecular biology [3,4]. The treatment approach of PTCL has customarily been based on the knowledge accumulated from diffuse large B cell lymphoma treatment. The standard first-line therapy still consists of cyclophosphamide, doxorubicin, vincristine, and prednisone (CHOP) or a CHOP-like regimen, although the outcome is poor, with frequent relapses and low 5-year overall survival and failure-free survival [5,6]. Routine introduction of targeted therapy for PTCL and other TCL types still requires the identification of solid predictor biomarkers that relate clinical and phenotypic variability to existing therapeutic options.

Thus, it is possible that, having molecularly characterized the individual TCL cases, we could identify potential candidates for targeted therapy. In this study, we integrated targeted deep sequencing with immunohistochemical analysis in a large cohort of 33 well-characterized T/NK-cell lymphoma/leukemia cell lines. This has provided insights into the specific molecular mechanisms underlying the pathogenesis of TCL and into the potential implications for future diagnosis and targeted therapy of TCL patients.

## Material and methods

### Cell lines

33 T/NK-cell lymphoma/leukemia cell lines were obtained from various sources (S1 Table). These included T-ALL (n = 20), ALCL (n = 5), CTCL (cutaneous T-cell lymphoma, n = 3), ATLL (adult T-cell lymphoblastic leukemia, n = 2), NK lymphoma subtypes (n = 2), and T-large granular lymphoma (T-LGL, n = 1) PTCL subtypes. Cell lines were cultured under basal conditions following the manufacturer’s instructions. All cell lines were purchased or authenticated before use and were tested for mycoplasma (MycoAlert™ mycoplasma detection kit; Lonza, Basel, Switzerland).

### Targeted amplicon-based enrichment and sequencing

16 genes were selected for sequencing. This set consisted of genes that are known potentially to play a role in tumorigenesis [7–20] (S2 Table). The gene panel was designed by Illumina Design Studio and comprised 547 amplicons, each of 170–190 bp. Libraries were prepared using the Illumina TruSeq Custom Amplicon Kit v1.5 and sequenced on a MiSeq sequencer (Illumina, San Diego, CA), following the manufacturer’s instructions. Variants were called using MiSeq Reporter and RUbioSeq [21], employing the default settings, and were visually inspected on IGV (www.broadinstitute.org/igv/). Variants were annotated with Variant Effect Predictor (GRCh37, http://grch37.ensembl.org/Tools/VEP). Known SNPs with an allelic frequency greater than 1% in public databases (dbSNP138, 1000 Genomes Project, Exome Sequencing Project, Exome Aggregation Consortium) were filtered out. In order to avoid false-positive calls, we performed duplicates with separate library preparation and sequencing in independent runs. Only variants called by both runs were considered.

### Tissue microarrays and immunostaining

Tissue microarrays (TMAs) were designed as described previously [22]using two 0.6-mm tissue cores per case, taken from formalin-fixed, paraffin-embedded archival tumor blocks. All immunostaining was done following standardized protocols. The panel of antibodies was chosen on the basis of their biological and clinical relevance in clinical classification and pathogenesis of TCL as well as with respect to their pharmacological implications (S3 Table). New antibodies were titrated with four or five dilutions (with an at least 2-fold difference between each) on the whole-mount tissue sections, according to the manufacturer’s recommendation. Each TMA was analyzed by at least two independent pathologists, who considered either the cytoplasmic or membranous staining intensity, or the percentage of positive nuclei. Specific thresholds are described in the S3 Table.

### Statistical analysis

Unsupervised hierarchical clustering with an average linkage algorithm was performed using Gene-E software v3.0.206 (www.broadinstitute.org/cancer/software/GENE-E). The Mann-Whitney U or Kruskal-Wallis tests were used to determine group differences. The chi-square or Fisher exact test was used as appropriate to determine associations between the presence or absence of markers. Statistical analyses were carried out using SPSS for Windows version 15 (Chicago, IL).

### Other resources and repositories

We consulted repositories with genomic data of TCL cell lines in order to ensure a broad landscape. Specifically, we unified genomic data from the CCLE (Cancer Cell Line Encyclopedia, http://www.broadinstitute.org/ccle), the COSMIC Cell Lines Project (http://cancer.sanger.ac.uk/cell_lines) [23], EGAS00001000268 [24] from the European Genome-Phenome Archive (https://www.ebi.ac.uk/ega/), and data from four exomes produced by our group in HH, HUT-78, MJ and Myla cell lines (S4 Table). Sequencing data have been deposited in the Sequence Read Archive (SRA) under accession reference SUB2029552.

## Results

### Variants identified by target enrichment and deep sequencing

**Fig 1:**
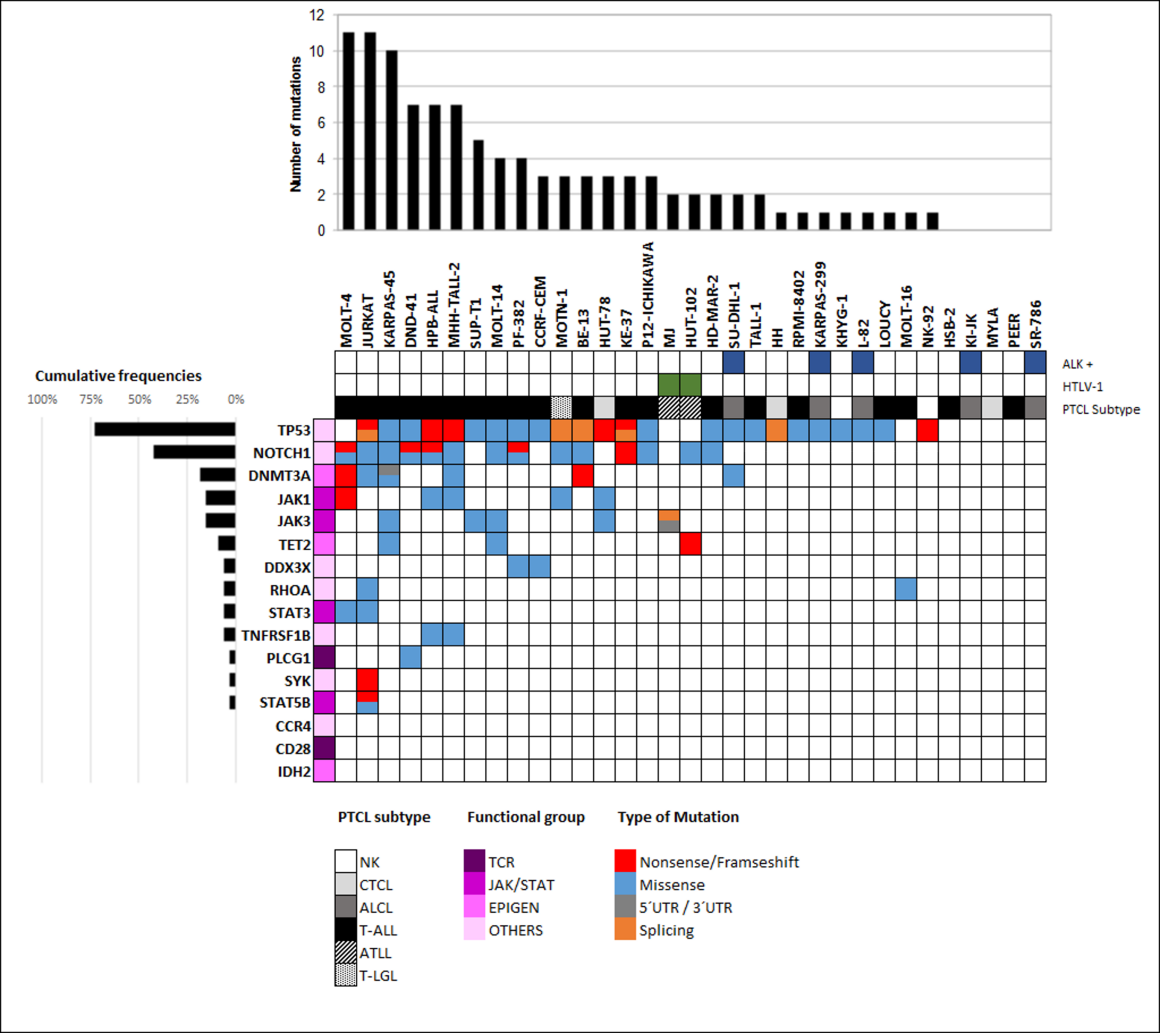
Mutational landscape of TCL cell lines. The results of targeted deep sequencing of 16 genes in 20 T-ALL (black), 5 ALCL (dark grey), 3 CTCL (medium grey), 2 NK (light grey), 2 ATLL (diagonal lines) and one T-LGL (dots) cell lines. Mutated genes (rows) are arranged in decreasing order of mutation frequency. Cell lines (columns) are arranged from left to right on the basis of their mutational frequency following gene ranking. HTLV-1-positive cell lines (green) and translocation t(2;5)(p23;q35) (ALK +, dark blue) are showed.

33 T/NK-cell lymphoma/leukemia cell lines were subjected to target amplicon-based enrichment and sequencing of the 16 selected genes (see details in S2 Table). On average, 91% of the amplicons in the panel studied had a depth of >100X, with 73% exceeding 500X. After conservative filtering, we validated 102 variants (S5 Table) in 15 genes from 30/33 samples (91%), including missense (74), frameshift (11), nonsense (8), splicing (7), and 3´/5´- UTR (2) variants (S5 Table and Fig 1). A mean of 3.1 SNVs per cell line (range: 0–11) was observed. We did not detect any SNVs in *CCR4*, *CD28* or *IDH2*.

**Fig 2:**
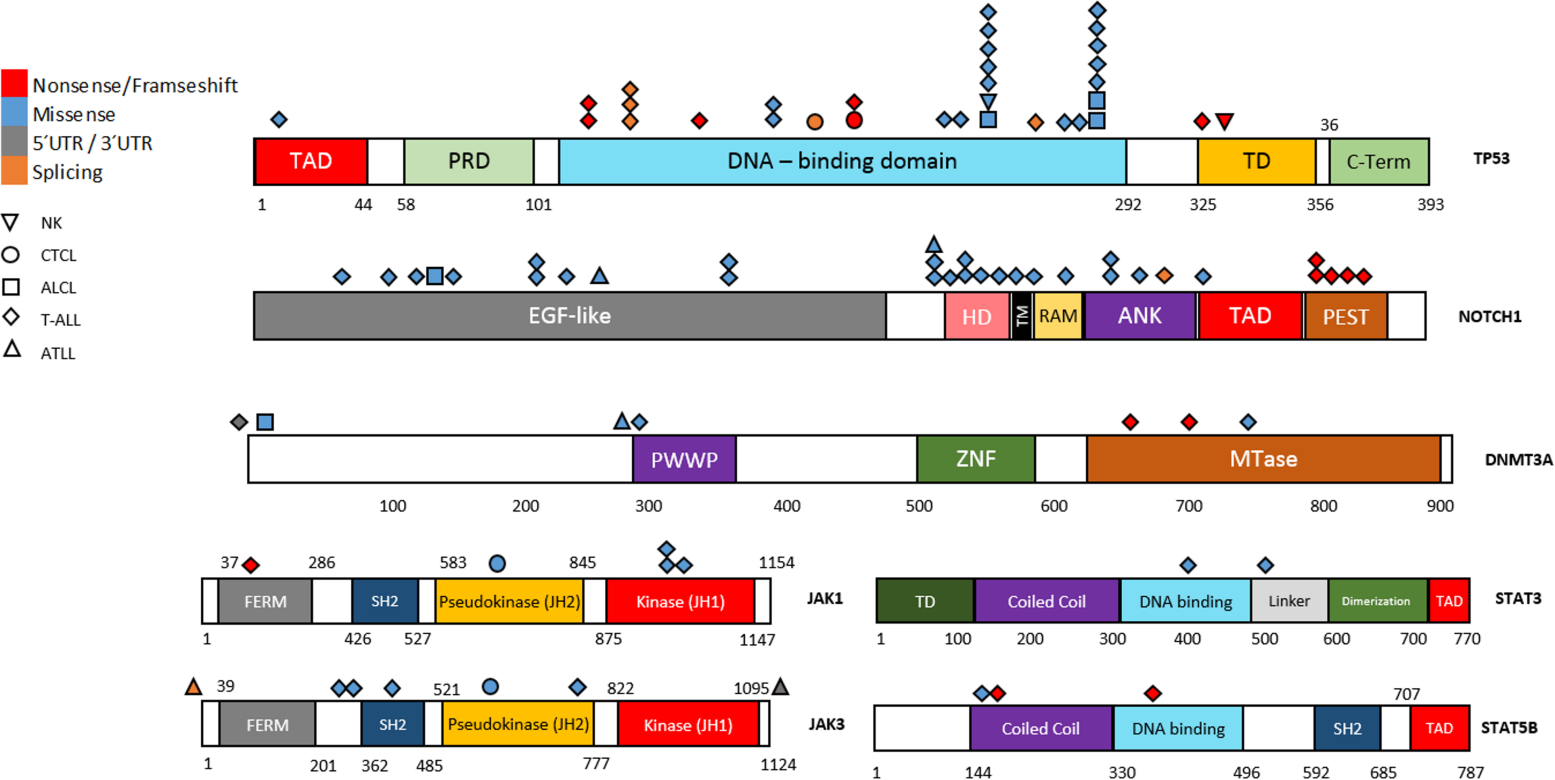
Mapping of variants in a TCL gene panel. Schematic of the alterations encoded by SNVs in *TP53*, *NOTCH1*, *DNMT3A*, *JAK1*, *JAK3*, *STAT3* and *STAT5B*. Type of variation and disease are represented by color and shape, respectively. TAD: transactivation domain; PRD: proline-rich domain; TD: tetramerization domain; C-term: C-terminal domain; HD: heterodimerization domain; TM: transmembrane domain; RAM: Rbp-associated molecule domain; ANK: ankyrin domain; PEST: proline (P), glutamic acid (E), serine (S), threonine (T) degradation domain; ZNF: zinc-finger domain; Mtase: methyltransferase domain.

*TP53*, *NOTCH1* and *DNMT3A* were altered in 72.7%, 42.4% and 18.2% of the cell lines, respectively. *TP53* harbored a large number of mutations, most of which were missense (21/33) and truncating mutations (7/33) (Figs 1 and 2A). Residues 248 and 273 were recurrently mutated, which produced different alterations at the nucleotide level. The P12-Ichikawa cell line carried a double-heterozygous mutation in the same nucleotide (c.743G>A/C; p.Arg248Gln/p.Arg248Pro) and seven cell lines had two or more *TP53* variants.

*NOTCH1* mainly harbored missense and truncating mutations (26 and 5 of 32 SNVs, respectively). We found more than one variant of *NOTCH1* per TCL cell line in six cell lines, with up to eight variants in MOLT4. *NOTCH1* SNVs were distributed throughout the whole gene. We found only truncating mutations in the PEST domain; these are known to lead to aberrantly prolonged signaling in the nucleus in this domain [25].

We detected 17 SNVs associated with the JAK/STAT pathway. *JAK3* and *JAK1* harbored seven and five variants, five and four of them being missense mutations, respectively. Three and two variants were found in *STAT5B* and *STAT3*, respectively. Interestingly, Jurkat harbored the three *STAT5B* and the one *STAT3* variants.

With respect to epigenetic-related genes, *DNMT3A* was the most frequently mutated gene with high diversity: we found seven variants, four of which were missense, two were truncating variants and one was located in the 5´UTR region. *TET2* had three missense variants and one truncating variant, whereas *IDH2* harbored no SNVs.

We found little variation in the other genes. We detected the same mutation (p.V385M) in HPB-ALL and MHH-TALL-2 in the *TNFRSF1B* gene. Two mutations were detected in *PLCG1* (both in the DND-41 cell line), *DDX3X and RHOA* and one was found in *SYK* (S5 Table).

### Variants identified by subtype

Among the cell lines, the T-ALL subtype carried the greatest frequency of SNVs (85/102, 4.25 SNVs per cell line). ATLL and CTCL both harbored 4/102 variants (2 and 1.33 SNVs per cell line, respectively) (Fig 1). We detected four and two variants (one SNV per cell line) in the ALCL and NK subtypes. *TP53* and *NOTCH1* mutations co-occurred in the T-ALL (11/20) and T-LGL cell lines (1/1), but not in any other subtype. NK cell lines featured solely *TP53* mutations. Mutations in genes involved in the JAK/STAT pathway were most frequently mutated in T-ALL. In this respect, four *JAK1* mutations, six *JAK3* mutations, and all *STAT3* and *STAT5B* mutations occurred in T-ALL cell lines. Only one mutation in the *JAK1* and in *JAK3* genes was detected in CTCL, which co-occurred in HUT-78. Similarly, the epigenetic genes *DNMT3A* and *TET2*, most of which were related to T-ALL, were found to be altered in these subtypes. Furthermore, *DNMT3A* was mutated in one ALCL and ATLL case each. Two novel *PLCG1* mutations were found in a single case of T-ALL (p.Q152H and p.D1199N).

### Expression of immunomarkers

**Fig 3:**
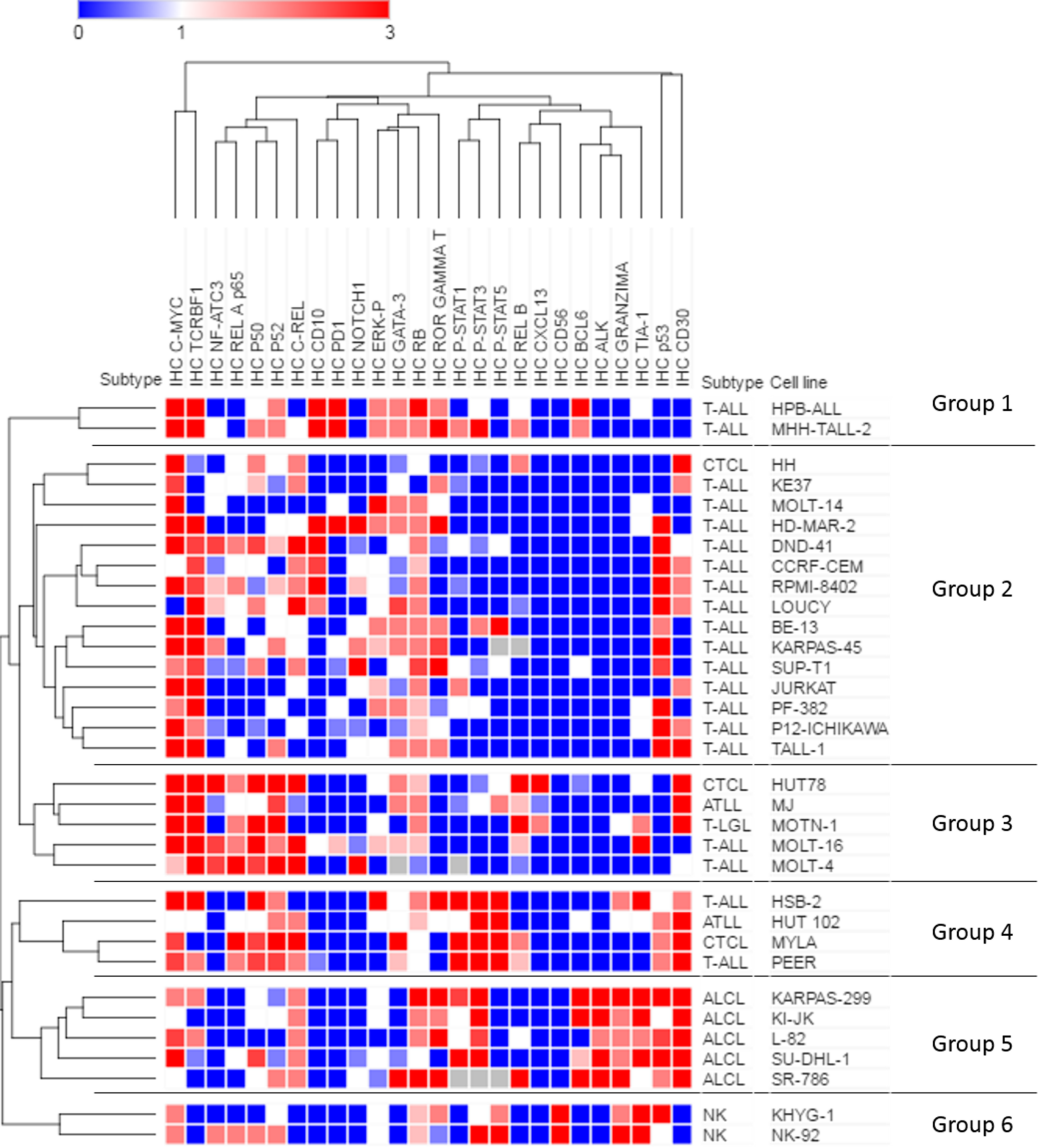
Unsupervised hierarchical clustering analysis with 26 immunomarkers. Each row represents a single cell line; each column represents a single immunomarker. Blue (score 0); white, weak immunostaining (score 1); light red (score 2); red, strong immunoreactivity (score 3); grey, missing data.

In order to identify a number of potentially deregulated disease actionable mechanisms, we used a set of 26 immunomarkers chosen not only on the basis of their biological and clinical relevance to clinical classification and pathogenesis of TCL, but also for their pharmacological implications (S3 Table). Hence, as shown in Fig 3, the NFKB pathway was activated in roughly half of the cell lines, both the canonical (p50/p65) and the non-canonical (p52/RelB), as indicated by the nuclear expression of the NFKB subunits. Nuclear NFAT was found in eight cases (24.2%), ERK and STATs proteins were activated in 30% and in 21–33% of cell lines, respectively, with STAT3 being the most frequent (Fig 3). The CD30 surface marker was expressed in 60.6% of cases, while CD10 and CD56 were detected in only 21.2% and 6.1%, respectively. Tumor suppressors p53 and RB were detected in 57.6% and 81.8% of cell lines, respectively. Notch1 was found in the nucleus (the active form) in five cases (15.2%) and its downstream target MYC was detected in 84.8%. GATA-3, ROR-gamma and TIA-1 showed positive expression in 15, 14 and 9 cell lines (45.5%, 42.4% and 27.2%), respectively (Fig 3).

### Unsupervised hierarchical clustering analysis of tissue microarray immunostaining

In order to classify our cases by specific immunohistochemical biomarkers, and to identify their potential association with pathogenesis, an unsupervised hierarchical clustering analysis (average linkage method) of the TMAs was undertaken. This produced a dendrogram with six well-defined clusters (Fig 3).

Most of the groups were defined by specific biomarkers. All groups clearly showed positive MYC and TCRBF1 expression, with the possible exception of group 6, which had limited TCRBF1 expression. Group 1 had differential positive PD1 expression alongside activated MAPK-ERK, GATA-3 and ROR-gamma-T. In group 2, the cluster featured broad RB staining (12/14) and heterogeneous expression of TP53, MAPK-ERK, NFAT and CD30. Group 3 showed the strongest activation of both canonical and non-canonical NFKB pathways, with positive expression of CD30 and NFAT in three of five cases. Group 4 showed characteristic constitutive activation of STAT 1, 3 and 5, with positive CD30 expression in all cases, along with the heterogeneous activation of the NFKB pathway in three of the four of cell lines. Group 5, formed exclusively of ALCL-ALK+ cell lines, was defined by strongly positive ALK, BCL6, CD30 Granzyme B and TIA-1 expression, together with STAT 3 activation. Group 6, comprised the two NK cell lines included in the study. They showed a typical NK signature positive for the expression of CD56, Granzyme B and TIA-1. This small group also showed activation of STAT 3 and 5. It is worth noting that under these circumstances, CTCL cell lines were dispersed into different groups.

Immunohistochemistry (IHC) scores were dichotomized to enable associations between markers to be determined (IHC score >1 = positive; IHC score ≤1 = negative) in all cell lines (S7 Table). Overall, the presence of canonical and of non-canonical NFKB pathway markers was significantly associated (p<0.05). Furthermore, the NFKB pathway was directly associated with NFAT (p65 RelA), CD30 (c-Rel) and GATA-3 (RelB), but inversely associated with ROR-gamma, p53 and RB (canonical NFKB), and MAPK-ERK (c-Rel). We found a positive association of the presence of ALK, Granzyme B, TIA-1 and BCL-6 with the activation of STAT 3 (p<0.05).

### Relation between IHC expression and mutational status

We analyzed the relation between mutational status and expression of specific immunomarkers. We subdivided *TP53* status into wild type, and missense and truncating mutation group. The expression of p53 was strongly associated with the presence of missense mutations compared with wild type and truncating mutations (p<0.001), (S1 Fig). However, we did not find any differences between *NOTCH1* status and Notch1 (S1 Fig) or MYC expression. In the case of MYC, 28/33 cell lines showed positive MYC expression, so we can conclude that MYC expression is not dependent on *NOTCH1* mutational status. Likewise, it is important to note that JAK mutations were not associated with the expression of their downstream targets. Only three of ten (30%) cell lines with mutated JAK showed STAT activation, as defined by nuclear staining. By contrast, ten out of 23 (43.5%) cell lines with JAK wild type showed STAT activation (S2 Fig).

## Discussion

Our growing knowledge about the molecular basis of T- and NK-cell lymphoma is leading to a better understanding of their pathogenesis and is helping refine the subclassification of TCL. Nevertheless, despite this progress, targeted therapy is still in a preliminary phase. The results presented here can help identify the more commonly deregulated mechanisms driving tumorigenesis in TCL, and provide a useful tool for analyzing the interaction between gene mutations and the activation of key survival pathways.

However, the panel of TCL cell lines tested has some limitations inherent to the difficulty of generating cell lines derived from particular T-cell lymphoma subtypes, notably AITL and PTCL-NOS. The panel is more representative for T-ALL, ATLL, CTCL, ALCL-ALK+ and NK subtypes. Despite these limitations, our results show that most T-cell lymphoma subtypes share mutations and activation of some essential pathways, such as JAK-STAT, NFKB, NFAT, chromatin regulation and others.

In this study we have examined 16 genes related to TCL pathogenesis [7–20], selected because of the presence of somatic mutations identified in previous studies, or due to their importance in TCL biology. We have identified 102 variants. A review of the data available in public repositories validated 64 of these SNVs (S6 Table) and identified 4 SNVs that were not picked up by our algorithm. On the other hand, 27 SNVs found in public repositories were not detected by our amplicon-based enrichment method. This discrepancy highlights how different methods may yield different results.

*TP53* was the mutated gene in our cell lines (72.7%). Truncating and missense mutations were correlated with low and high levels of p53 expression, respectively. The *NOTCH1* gene was also frequently mutated, with five truncating mutations located in the PEST domain. Only the MOLT-4 cell line showed a high level of expression of Notch1; it was not expressed in the other cell lines (DND-41, HPB-ALL, KE-37 and PF-382). Whereas KE-37 cell line harbored only one mutation in the PEST domain, the DND-41, HPB-ALL, MOLT-4 and PF-382 cell lines were also found to be mutated in the HD domain. The MOLT-4 cell line harbored eight mutations in *NOTCH1*, localized in different domains from the PEST and HD domain. It has been reported that truncating mutations in the PEST domain lead to aberrantly prolonged signaling in the nucleus, but are only functional in the presence of Notch ligands [25]. Mutations in the HD domain, which comprises exons 26 and 27, destabilize the interaction between the N- and C-terminal HD subunits, resulting in increased signaling through either ligand-independent or ligand-hypersensitive activation of Notch1, or in the displacement of the processing site for ADAM cleavage, allowing for constitutive ligand-independent metalloprotease processing [25]. Mutations in other domains need to be functionally elucidated. Therefore, understanding the complexity and consequences of Notch activation is critical for defining optimal therapeutic strategies targeting the Notch pathway.

Mutations in the JAK/STAT pathway have been reported in PTCL patients [11,12,20]. We found 17 different mutations in 12 cell lines, which enabled us to detect mutations in *JAK1* and *JAK3* genes in 27.3% of the cell lines analyzed. HUT-78 showed mutations in JAK1 and JAK3 pseudokinase domains [20] and MOLT-14 in JAK1 the pseudokinase domain. Mutations in these domains have been widely reported and are usually associated with increased downstream signaling in some hematological malignancies as well as in solid tumors. Thus, it has been shown that JAK pseudokinases are autoinhibitory domains that keep the kinase domain inactive until receptor dimerization stimulates transition to an active state. Nonetheless, these three cell lines showed no activation of STAT proteins. This lack of a genotype-phenotype correlation between mutations in the pseudokinase domain and STAT expression (S1 Fig) can be explained by the basal conditions (e.g., without cytokines) in which the cells were cultured [26]. Mutations in the *JAK1* kinase domain were found in three cases (HPB-ALL, MHH-TALL-2 and MOTN-1 cell lines). It is important to note that the HPB-ALL and MHH-TALL-2 cell lines shared the same mutation (p.Q966V), but STAT was activated only in the MHH-TALL-2 cell line. The molecular significance of these mutations is not easy to interpret, since they could act in a receptor-dependent or independent manner with respect to activation. Therefore, although JAK inhibitors (JAKis) constitute a new therapeutic option for the treatment of PTCL patients [20,26], further studies are needed to elucidate the relation between mutations and the activation of the JAK/STAT pathway as well as the mechanisms of JAKi resistance.

Mutations of epigenetic regulators are so common in PTCL that they constitute one of the largest groups of mutation, including those affecting the splicing machinery, signaling pathways and transcription factors [2]. Mutations in *DNMT3A* and *TET2* were found in 18.2% and 9.1% of our panel of cell lines, respectively. *DNMT3A* encodes a protein that catalyzes methylation and demethylation of DNA, depending on the microenvironment conditions [27]. The specific relevance of *DNMT3A* mutations to the cancer phenotype has not been explored, except for p.R882 mutations, which predict poor prognosis in acute myeloid leukemia [28,29]. TET family proteins are known to play critical roles in DNA demethylation by converting 5-mC to 5-hydroxymethylcytosine (5-hmC) in α-KG-dependent and a Fe (II)-dependent manner [30]. Mutations that disrupt the catalytic domain or lead to a truncated TET2 have been linked to the development of hematological malignancies [31]. In fact, several leukemia and lymphoma disorders have a *TET2* that is mutated at notably high frequencies (chronic myelomonocytic leukemia: 35–50%; AITL: 50–80%; PTCL-NOS: 40–50%) [32–37]. Some epigenetic drugs, such as vorinostat, belinostat and romidepsin, have been positioned as a second line for TCL treatment, and have produced improved response rates.

This study found two mutations in *PLCG1* (encoding p.Gln152His and p.Asp1199Asn), both of which were present in a T-ALL cell line, DND-41. Recently, two hot-spot *PLCG1* mutations (encoding p.Ser345Phe and p.Ser520Phe) that enhance PLCƔ activity have been reported in T-cell lymphoma [19,38]. *PLCG1* encodes phospholipase CƔ1 (PLCƔ1), a key regulator of proximal TCR signaling [38]. Interestingly, NFAT expression was positive in the cells harboring *PLCG1* mutations, suggesting that these mutations may promote deregulated activation of downstream PLCƔ1 signaling. This activation may support the idea that specific targeting of PLC downstream signaling, like tacrolimus, which acts as a calcineurin inhibitor, could be a therapeutic option for the treatment of patients with mutations in *PLCG1*.

Two *RHOA* mutations were detected, both of them in T-ALL. Several research groups have found frequent *RHOA* mutations, specifically the p.G17V mutation, in AITL and PTCL patients [37,39]. Interestingly this p.G17V mutation appears to act similarly to well-characterized dominant negative mutations of *RHOA*, rather than as an activating mutation. Although none of the mutations found in our study corresponds to the p.G17V variant, it is important to note that both cells lines in our study that harbor *RHOA* mutations showed robust MYC expression. In this context, it has been reported that there is cross-regulation between MYC and RhoA activation [40].

From a therapeutic perspective, our results highlight important disease mechanisms that have the potential to serve as targets for therapy. In this regard, the immunohistochemical analysis identified an activated NFKB pathway in about 62% of TCL cell lines (Fig 3). Recently, Odqvist and colleagues reported worse overall survival in PTCL patients associated with nuclear expression of classical or alternative NFKB components, implying that NFKB-inducing kinase (NIK) silencing could be an effective target for abrogating the NIK-dependent NFKB activation [41]. The number of NIK inhibitors currently known is limited. A preclinical study with ALK-negative ALCL patient cells [42] and CTCL cell lines [43] reported the potential for the effective use of bortezomib, but a phase II study in refractory ATLL patients was cancelled because single-agent activity did not produce significant improvements in patients [44]. NIK and IKK inhibitors may be promising agents in T-cell lymphomas with an activated NFKB pathway, but further studies and clinical trials are needed to evaluate the real potential of these agents in single and combined usage.

The second most frequently activated pathway in cell lines was JAK/STAT (42.4%), making the blockade of this pathway a promising means of treating TCL patients. Ruxolitinib has been demonstrated to inhibit CTCL cell line proliferation at micromolar concentrations [20] and clinical trials are now ongoing (www.clinicaltrials.gov; accessed September 2016) in T-cell lymphomas and other hematological malignancies. Tofacitinib has been shown to inhibit JAK3 in CTCL [45] but other JAK inhibitors such as momelotinib, baricitinib or filgotinib have not been tested in TCL. Although few preclinical and clinical data are available, STAT3 inhibitors, which seem to have a low toxicity profile [46], are other emerging targets.

Unsupervised hierarchical clustering identified six groups on the basis of their expression profile. We can propose a targeted therapy that takes into account the mutational background of each group (S2 Fig). Group 1 had a differentially positive PD1 expression and activated MAPK-ERK. Given this, anti-PD1 and ERK inhibitors could constitute an effective therapy for this group. A recent phase I study noted a response rate of 17% with nivolumab treatment [47]. Group 2, mainly composed of T-ALL cell lines, was complex because of the heterogeneous expression of immunomarkers, so different approaches should be adopted to treat such patients. Group 3 exhibited the strongest activation of both canonical and non-canonical NFKB pathways, with strong expression of CD30, so drugs reducing NFKB activation and anti-CD30 may be good options for therapy. Interestingly, Group 4 showed activation of STAT 1, 3 and 5, with positive expression of CD30 in all cases. Anti-CD30 and JAKi therapy could be a treatment option for this group. Group 5, comprising the ALCL-ALK+ cell lines, was strongly positive for ALK, BCL6, CD30 and STAT3, so the treatment options could include the use of anti-CD30 antibody and ALK and JAK inhibitors. In fact, brentuximab vedotin, an anti-CD30 antibody, has recently been approved to treat ALCL patients [48]. On the other hand, the ALK inhibitor alectinib was tested in the ALCL-ALK+ cell line KARPAS-299 [49], in which it showed potent efficacy in a KARPAS-299 mouse xenograft. Group 6 comprised only the two NK cell lines included in the study. As recently reported [50], JAKi could be a new option for treating this lymphoma subtype.

In conclusion, the study identifies commonly deregulated pathways and genes in TCL, including JAK/STAT, NOTCH, NFKB and chromatin conformation. Activation of these pathways is somehow the consequence of somatic mutation and other causes. Our findings may help in the development of preclinical models for the evaluation of new targeted drugs.

## Supporting information

S1 Table Cell lines used in the study. (XLSX)

S2 Table List of genes sequenced by amplicon-based methodology in cell lines. (XLSX)

S3 Table Antibodies used in immunohistochemical analysis. (XLSX)

S4 Table Public data consulted to validate our 16-gene panel. (XLSX)

S5 Table List of variants found in our panel of cell lines. (XLSX)

S6 Table List of variants found in public data. (XLSX)

S7 Table Association between immunomarkers. Probabilities of chi-square or Fisher exact tests for tests of association between pairs of the 26 immunomarkers used. Green and orange indicate positive and negative associations, respectively.
(XLSX)

S1 Fig Genotype-phenotype associations. a) Mutational status of *TP53* was defined as Nonsense/Frameshift (n = 10, blue), wild type (WT, n = 9, white) and missense (n = 15, red). Mutational status of *NOTCH1* was defined as wild type (n = 20, White) and *mutated* (n = 14, red). Mutational status of JAK was defined to be *JAK1* and/or *JAK3* wild type (n = 23, white) or mutated (n = 11, red). The immunomarkers p53, NOTCH1 and STATs, are indicated in color as in Fig 3. STATs was defined as the mean of p-STAT1, p-STAT3 and p-STAT5. b) Mean of immunomarkers with respect to mutational status. Error bars indicate the SEM (standard error of mean).
(TIF)

S2 Fig Mutational landscape of TCL cell lines grouped by unsupervised hierarchical clustering. (TIF)
