# Supplementary figures and images for: Molecular basis of targeted therapy in T/NK-cell lymphoma/leukemia: A comprehensive genomic and immunohistochemical analysis of a panel of 33 cell lines
Source: PLoS One. 2017 May 15;12(5):e0177524. doi: 10.1371/journal.pone.0177524 (PMC5432176; doi:10.1371/journal.pone.0177524)

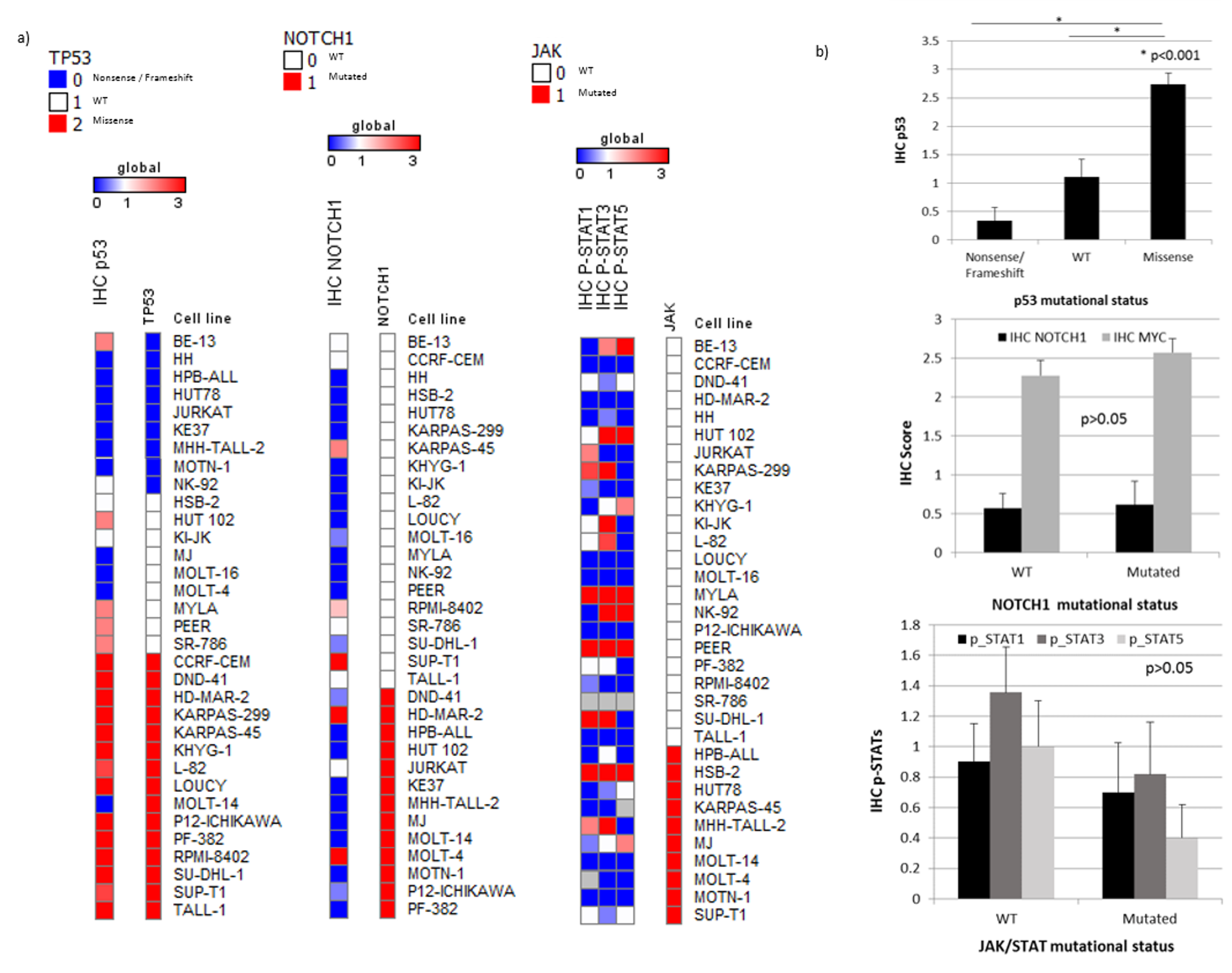

Supplement: S1 Fig — a) Mutational status of TP53 was defined as Nonsense/Frameshift (n = 10, blue), wild type (WT, n = 9, white) and missense (n = 15, red). Mutational status of NOTCH1 was defined as wild type (n = 20, White) and mutated (n = 14, red). Mutational status of JAK was defined to be JAK1 and/or JAK3 wild type (n = 23, white) or mutated (n = 11, red). The immunomarkers p53, NOTCH1 and STATs, are indicated in color as in Fig 3. STATs was defined as the mean of p-STAT1, p-STAT3 and p-STAT5. b) Mean of immunomarkers with respect to mutational status. Error bars indicate the SEM (standard error of mean). (TIF) [file pone.0177524.s008.tif]

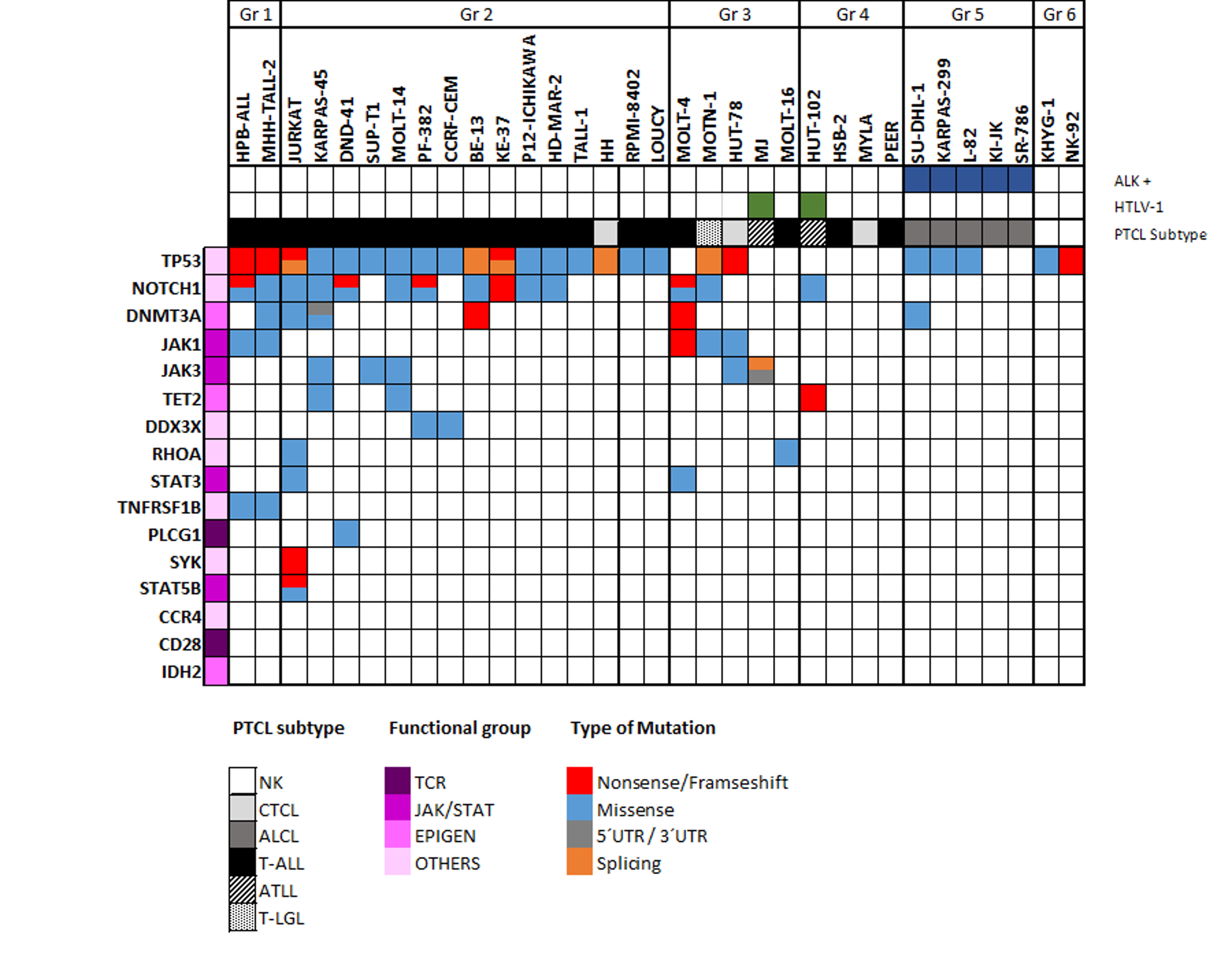

Supplement: S2 Fig — (TIF) [file pone.0177524.s009.tif]
